# A perovskite oxide with high conductivities in both air and reducing atmosphere for use as electrode for solid oxide fuel cells

**DOI:** 10.1038/srep31839

**Published:** 2016-08-22

**Authors:** Rong Lan, Peter I. Cowin, Sivaprakash Sengodan, Shanwen Tao

**Affiliations:** 1 School of Engineering, University of Warwick, Coventry CV4 7AL, UK; 2 Department of Chemical and Process Engineering, University of Strathclyde, Glasgow G1 1XJ, UK; 3 Department of Chemical Engineering, Monash University, Clayton, Victoria 3800, Australia

## Abstract

Electrode materials which exhibit high conductivities in both oxidising and reducing atmospheres are in high demand for solid oxide fuel cells (SOFCs) and solid oxide electrolytic cells (SOECs). In this paper, we investigated Cu-doped SrFe_0.9_Nb_0.1_O_3−δ_ finding that the primitive perovskite oxide SrFe_0.8_Cu_0.1_Nb_0.1_O_3−δ_ (SFCN) exhibits a conductivity of 63 Scm^−1^and 60 Scm^−1^ at 415 °C in air and 5%H_2_/Ar respectively. It is believed that the high conductivity in 5%H_2_/Ar is related to the exsolved Fe (or FeCu alloy) on exposure to a reducing atmosphere. To the best of our knowledge, the conductivity of SrFe_0.8_Cu_0.1_Nb_0.1_O_3−δ_ in a reducing atmosphere is the highest of all reported oxides which also exhibit a high conductivity in air. Fuel cell performance using SrFe_0.8_Cu_0.1_Nb_0.1_O_3−δ_ as the anode, (Y_2_O_3_)_0.08_(ZrO_2_)_0.92_ as the electrolyte and La_0.8_Sr_0.2_FeO_3−δ_ as the cathode achieved a power density of 423 mWcm^−2^ at 700 °C indicating that SFCN is a promising anode for SOFCs.

Solid oxide fuel cells (SOFCs) are electrochemical devices used to convert chemical energy into electricity with a very high efficiency^1^. Symmetrical fuel cells have the potential application to be used as reversible SOFCs, which can operate in both fuel cell and electrolyser modes. In a recent report, it was found that a much better stability can be achieved by SOFCs which operate in both SOFC and solid oxide electrolytic cell (SOEC) modes (reversible SOFC)^2^. For reversible SOFCs, the use of a symmetrical electrode such as (La_0.75_Sr_0.25_)Cr_0.5_Mn_0.5_O_3−δ_ (LSCM) would be an ideal solution^3^,^4^,^5^. An essential requirement of the electrode material is that it must exhibit a high conductivity in both air and fuel conditions at the fuel cell operating temperature. Therefore, electrode materials exhibiting high conductivities in both oxidising and reducing atmospheres are in high demand for solid oxide fuel cells (SOFCs) and solid oxide electrolytic cells (SOECs), particularly for symmetrical SOFCs.

There are plenty oxide materials which exhibit high conductivity in air. The real challenge is to identify a good stable oxide anode material exhibiting high conductivity in a reducing atmosphere^5^,^6^. Several redox stable mixed oxide-ion electronic conductors have been developed as ceramic anode materials for SOFCs, such as (La_0.75_Sr_0.25_)Cr_0.5_Mn_0.5_O_3−δ_ (LSCM)^7^, Sr_2_MgMoO_6−δ_ (SMMO)^8^, La_4_Sr_8_Ti_11_Mn_0.5_Ga_0.5_O_37.5_ (LSTMG)^9^, La_0.8_Sr_0.2_Sc_0.2_Mn_0.8_O_3−δ_ (LSSM)^10^, La_0.7_Ca_0.3_CrO_3−δ_^11^, Sr_2_Fe_1.5_Mo_0.5_O_6−δ_^12^,^13^; Pr_0.8_Sr_1.2_(Co,Fe)_0.8_Nb_0.2_O_4+δ_/CoFe alloy^14^, PrBaMn_2_O_5+δ_^15^, La_0.33_Sr_0.67_Ti_0.33_Mn_0.67_O_3−δ_ (LSTM)^16^, Ce_0.6_MN_0.3_Fe_0.1_O_2_-La_0.6_Sr_0.4_Fe_0.9_Mn_0.1_O_3_ (CMF-LSFM)^17^. All of these oxides or composite anodes are redox stable or redox reversible. However, except for La-doped SrTiO_3_, the conductivity at the anode is typically below 30 S/cm. The anode conductivity needs to be further improved, particularly for tubular SOFCs with a long pathway for electrons. The conductivity of La-doped SrTiO_3_ in a reducing atmosphere is high if pre-reduced at a high temperature. However, the conductivity of doped SrTiO_3_ in air is generally less than 0.1 S/cm^6^,^9^,^18^. In reported perovskite oxides, besides LSCM, The double perovskite Sr_2_Fe_1.5_Mo_0.5_O_6−δ_ (SFMO), was demonstrated to be a good electrode for symmetrical SOFCs^12^,^13^. However, the conductivity of SFMO under the SOFC anode environment is not very high thus further improvements are required^13^. On the other hand, a promising family of redox stable anodes for SOFCs is B-site doped SrFeO_3−δ_. It has been reported that SrFe_1-x_Ti_x_O_3−δ_ where x = 0.3, 0.4 combined with Ce_0.9_Gd_0.1_O_2−δ_ performs well as an anode for SOFCs^19^. It was found that SrFe_0.9_Ti_0.1_O_3−δ_ is redox stable with a conductivity of 2.53 S/cm at 600 °C in a reducing atmosphere^20^. Anikina *et al*. investigated the conductivity of SrFe_1−x_Nb_x_O_3−δ_, where x = 0.05, 0.1, 0.2, 0.3, 0.4 and it was found SrFe_0.9_Nb_0.1_O_3−δ_ exhibits the highest conductivity in a reducing atmosphere. However, the conductivity is still below 1 S/cm at temperatures below 800 °C^21^. In this study, we re-investigated the conductivity of SrFe_0.9_Nb_0.1_O_3−δ_ finding that its conductivity in a reducing atmosphere was ~30 S/cm which is much higher than the reported values. It was also found that partial replacement of Fe by Cu in SrFe_0.9_Nb_0.1_O_3−δ_ can further increase the conductivity in a reducing atmosphere. The perovskite oxide SrFe_0.8_Cu_0.1_Nb_0.1_O_3−δ_ (SFCN) exhibits a conductivity of 63 Scm^−1^ and 60 Scm^−1^ at 415 °C in air and 5%H_2_/Ar respectively. To the best of our knowledge, the conductivity of SrFe_0.8_Cu_0.1_Nb_0.1_O_3−δ_ in a reducing atmosphere is the highest among reported oxide anodes for SOFCs which also exhibit a high conductivity in air. A SOFC using SrFe_0.8_Cu_0.1_Nb_0.1_O_3−δ_ as the anode and La_0.8_Sr_0.2_FeO_3−δ_ as the cathode has been fabricated with a good performance in hydrogen achieved at temperatures below 700 °C.

## Structure of New Oxides SrFe_0.9−x_Cu_x_Nb_0.1_O_3−δ_ (x = 0–0.4)

**Figure 1:**
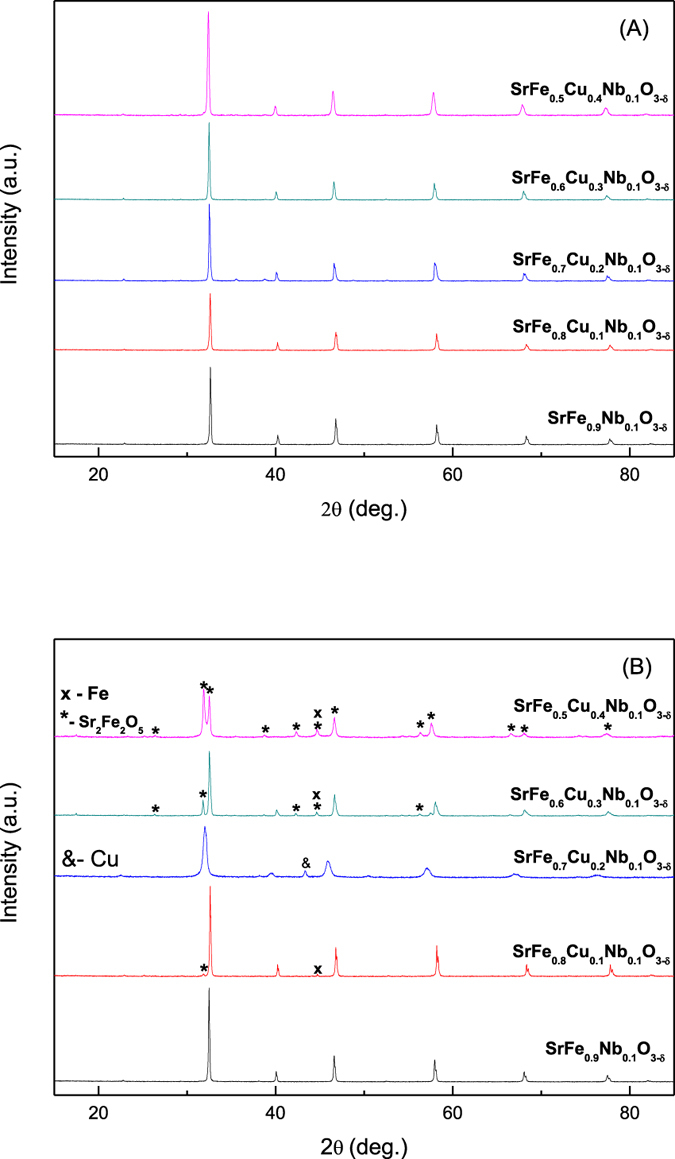
Room temperature X-ray diffraction patterns of SrFe_0.9−x_Cu_x_Nb_0.1_O_3−δ_ (x = 0–0.4) (x = 0–0.4) after obtained in air (**A**) and after further reduction in 5%H_2_/Ar at 700 °C for 10 hours (**B**).

**Figure 2:**
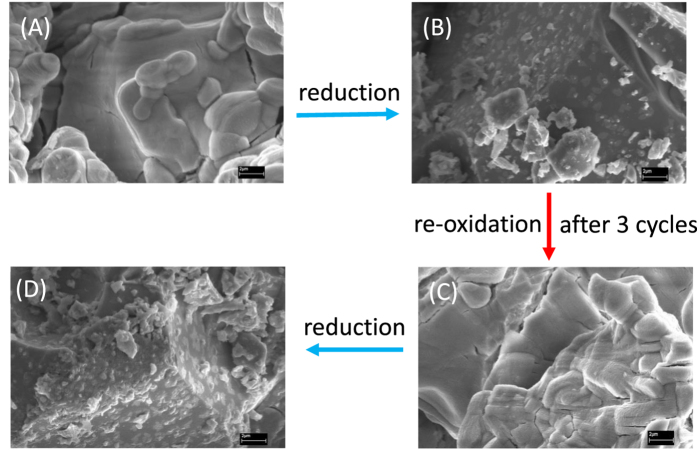
SEM pictures of sample SrFe_0.8_Cu_0.1_Nb_0.1_O_3−δ_ powders after obtained in air (**A**); after further reduction in 5%H_2_/Ar at 700 °C for 10 hours (**B**); after further xoidise in air at 1300 °C for 15 hours then reduce at 700 °C in 5%H_2_/Ar for 10 hours (3 cycles with final step of reoxidation) (**C**) and futher reduction in 5%H_2_/Ar at 700 °C for 10 hours (**D**).

X-ray diffraction of SrFe_0.9−x_Cu_x_Nb_0.1_O_3−δ_ (x = 0–0.4) showed that it exhibited a single phase cubic perovskite structure (SG: *Pm-3m*) for all compounds (Figure S1). The XRD pattern of the SrFe_0.8_Cu_0.1_Nb_0.1_O_3−δ_ sample is shown in Fig. 1A. The increase in the copper content significantly reduced the sintering temperature of the material, with the formation of a single phase perovskite structure that was not observed for SrFe_0.4_Cu_0.5_Nb_0.1_O_3−δ_. Reitveld refinement of the structure using GSAS^22^ demonstrated a pseudo-linear increase in the lattice parameters with increased copper doping up to SrFe_0.6_Cu_0.3_Nb_0.1_O_3−δ_ (Figure S3 and Table S2). The increase in lattice parameters with increasing copper content can be attributed to the larger ionic radius of copper compared to iron (Cu^2+^0.73 Å, Fe^3+^0.645 Å, Fe^4+^0.585 Å)^23^. Samples SrFe_0.6_Cu_0.3_Nb_0.1_O_3−δ_ and SrFe_0.5_Cu_0.4_Nb_0.1_O_3−δ_ exhibit similar lattice parameters whilst no impurity peaks can be observed on the XRD patterns, this is thought to be due to the proximity of the solid solution limit. Therefore the limit for achieving single phase in the SrFe_0.9−x_Cu_x_Nb_0.1_O_3−δ_ series lies at x ≤ 0.5. The SEM picture of SrFe_0.8_Cu_0.1_Nb_0.1_O_3−δ_ prepared by the solid state reaction method is shown in Fig. 2A.

Thermogravimetric analysis of SrFe_0.9−x_Cu_x_Nb_0.1_O_3−δ_ (x = 0–0.4) in air showed a minor total loss in weight for all compounds, between 0.2 wt% and 0.5 wt%, with no observable trend on increasing the dopant level, (Figure S3A). Accelerated weight loss was observed for all compounds on heating between 500 and 800 °C, with the weight loss noted to be reversible upon cooling. This acceleration in weight loss is likely to be the result of oxygen loss through high temperature reduction. Differential scanning calorimetry, Figure S3B, exhibits a reversible transition for all copper doped compounds, between 600 °C and 670 °C on heating and between 670 °C and 590 °C on cooling which could be related to high temperature phase transition^24^.

## Conductivity of New Oxides SrFe_0.9−x_Cu_x_Nb_0.1_O_3−δ_ (x = 0–0.4) in Air

**Figure 3:**
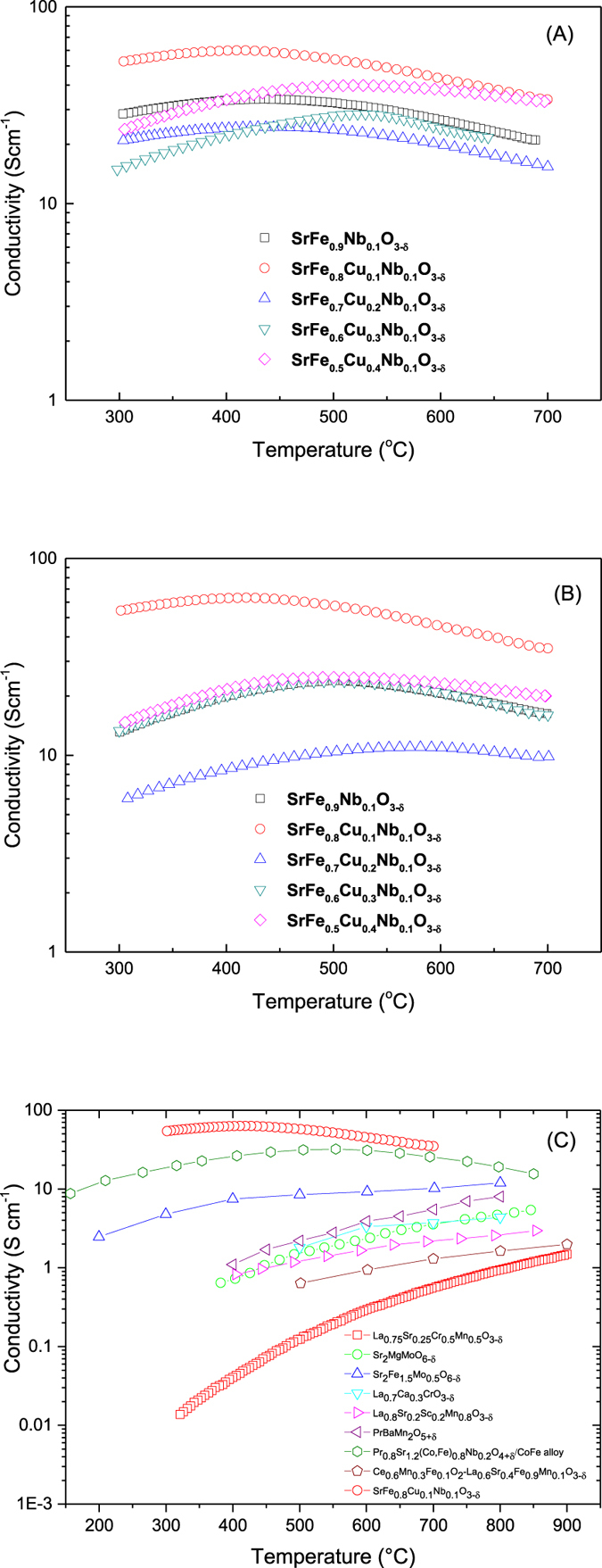
Conductivity of SrFe_0.9−x_Cu_x_Nb_0.1_O_3−δ_ (x = 0–0.4) in air (**A**) 5% H_2_/Ar (**B**) and comparison of conductivities of reported anode materials in H_2_ or 5%H_2_ (**C**).

The conductivity of the electrode materials is a key parameter to consider when evaluating their use in SOFCs. Whilst minimal copper doping, forming SrFe_0.8_Cu_0.1_Nb_0.1_O_3−δ_, elicits a significant increase in conductivity in air over SrFe_0.9_Nb_0.1_O_3−δ_, additional copper doping is observed to reduce the conductivity of the materials, although increasing dopant levels do not elicit a linear response in the reduction in conductivity (Fig. 3A). The introduction of Cu^2+^ dopant is expected to increase the average charge of iron in the sample with the proportion of Fe^4+^ ions, increasing the number of charge carriers and thus, in theory, increasing the conductivity^25^. Assuming that the copper dopant does not directly contribute to electronic conduction, the increase in the charge carrier concentration is only proportional to the iron content of the compound, which reduces with increasing Cu^2+^ dopant concentration. Thus at higher Cu^2+^ dopant concentrations the increase in charge carriers through the average charge of iron increasing is outweighed by the reduction in charge carriers through the reduction of the iron content leading to reduced conductivity. On the conductivity curves, a semiconductor-metal transition was observed for all compounds, with an increase in the transition temperature noted with increasing copper dopant levels (Fig. 3A). This transition has been observed previously for strontium ferrites, with Poulsen *et al*.^26^ suggesting that compound reduction at high temperature, resulting in a reduction in the charge carriers, was the cause of the transition. A pseudo-linear reduction in the oxygen content of strontium ferrite in air above 400 °C with increasing temperature, which would appear to confirm compound reduction at these temperatures, was observed previously^27^. The increase in the conductivity with increasing temperature was determined by Patrakeev *et al*. to be offset by the reduction in charge carrier concentration, causing an overall reduction in the electronic conductivity with increasing temperature, resulting in the pseudo-metallic behaviour^28^.

## Stability of New Oxides SrFe_0.9−x_Cu_x_Nb_0.1_O_3−δ_ (x = 0–0.4)

In order to investigate the stability of SrFe_0.9−x_Cu_x_Nb_0.1_O_3−δ_ (x = 0–0.4) in a reducing atmosphere, STA analyses in 5%H_2_/Ar was carried out on the samples. The observed weight loss upon reduction of SrFe_0.9−x_Cu_x_Nb_0.1_O_3−δ_ (x = 0–0.4) varies between 2.6% and 3.3%, with no observed trend with increasing dopant concentration (Figure S4A). Differential scanning calorimetry exhibits non-reversible transitions on heating for all compounds between 600 °C and 670 °C, associated with cationic reduction (Figure S4B)^20^. With the increase of copper dopant, the exothermic peaks happened at lower temperature indicating the reduction at lower temperature thus they are likely less stable, this has been confirmed by the XRD study (Fig. 1B). After reducing in 5%H_2_/Ar at 700 °C for 10 hours, it was found that the Cu-free sample SrFe_0.9_Nb_0.1_O_3−δ_ was redox stable. For sample SrFe_0.8_Cu_0.1_Nb_0.1_O_3−δ_, the majority of the phase is perovskite whilst an extra peak at ~45 degree was observed which belongs to the strongest (110) peak of α-Fe (PDF: 6–696) after the reduction at 700 °C^29^. The formation of a Fe-rich FeCu alloy cannot be ruled out but the Cu content must be very low otherwise the peak should shift to a higher d-spacing. The exsolution of metal particles is further confirmed by SEM observation where a small particle of iron was exsolved on the surface after the reduction (Fig. 2B). The very weak peak at ~32.5° could be a peak for a solid solution based on Sr_2_Fe_2_O_5+δ_ parent phase^30^. When x is increased to 0.2, the majority of sample SrFe_0.7_Cu_0.2_Nb_0.1_O_3−δ_ is possibly Sr_2_Fe_2_O_5+δ_ solid solution while an extra peak at ~43° was observed which could be the strongest peak of Cu (*Fm-3m*)^31^. Again, formation of a Cu-rich FeCu alloy is also possible with the presence of Fe at the B-site of the perovskite phase. At x = 0.3, 0.4, Sr_2_Fe_2_O_5+δ_ based solid solution is the major phase with the Fe or Fe-rich alloy also present. The exsolution of metal seems strongly related to the composition, which will require further investigation. To exam whether the exsolution of metal is reversible, the reduced SrFe_0.8_Cu_0.1_Nb_0.1_O_3−δ_ sample was re-oxidised in air at 1300 °C for 15 hours then further reducing in 5%H_2_/Ar at 700 °C for 10 hours and stop at the re-oxidation stage after 3 cycles. It was observed that the exsolved metal was ‘adsorbed’ back after re-oxidation ii air at high temperature (Fig. 2C) while it was exsolved out after further reduction in 5%H_2_/Ar at 700 °C for 10 hours (Fig. 2D). This indicate the process for exsolved metal is reversible as observed in other oxides^32^,^33^.

## Conductivity of New Oxides SrFe_0.9−x_Cu_x_Nb_0.1_O_3−δ_ (x = 0–0.4) in 5%H_2_/Ar

To measure the conductivity in a reducing atmosphere, the SrFe_0.9−x_Cu_x_Nb_0.1_O_3−δ_ pellets were coated with silver electrodes on both sides then reduced in 5%H_2_/Ar at 700 °C for 10 hours (Fig. 3B). The conductivity was measured in 5%H_2_/Ar on cooling. The highest conductivity for the samples measured was found to be for the SrFe_0.8_Cu_0.1_Nb_0.1_O_3−δ_ sample with a conductivity of about 30–60 S/cm, in a reduced atmosphere. This is possibly due to the presence of exsolved Fe particles leading to increased electronic conductivity thus the total conductivity was also high. The lowest conductivity was observed for sample SrFe_0.7_Cu_0.2_Nb_0.1_O_3−δ_, due to the Sr_2_Fe_2_O_5+δ_ based solid solution being the major phase. This indicates that the conductivity of Sr_2_Fe_2_O_5+δ_ based solid solution is lower than the primitive perovskite oxide based on SrFeO_3−δ_. When x increased to 0.3 and 0.4, the intensity of the peak at ~45 ° which represents the exsolved Fe is obviously stronger than that for sample SrFe_0.8_Cu_0.1_Nb_0.1_O_3−δ_ (Fig. 1B) even though the major phase was the Sr_2_Fe_2_O_5+δ_ based solid solution. The conductivity of SrFe_0.6_Cu_0.3_Nb_0.1_O_3−δ_ is comparable to that for the Cu-free sample SrFe_0.9_Nb_0.1_O_3−δ_ whilst the conductivity of sample SrFe_0.5_Cu_0.4_Nb_0.1_O_3−δ_ is slightly higher (Fig. 3B) due to higher concentration of exsolved Fe (or Fe-rich alloy). In the investigated oxides, in terms of both redox stability and conductivity, sample SrFe_0.8_Cu_0.1_Nb_0.1_O_3−δ_ is the best.

For comparison, the conductivity of sample SrFe_0.8_Cu_0.1_Nb_0.1_O_3−δ_ in a reducing atmosphere is plotted together with the reported best oxide anodes for SOFCs (Fig. 3C). It is the highest among the reported materials with reasonably high conductivity in air and a conductivity two times greater than that of the Pr_0.8_Sr_1.2_(Co,Fe)_0.8_Nb_0.2_O_4+δ_/CoFe alloy^14^. The conductivities of all other reported oxide anode materials in a reducing atmosphere are lower than 30 S/cm. This is clearly shown when the conductivity is plotted at absolute value (Figure S5). High conductivity is very important for SOFCs due to some designs involving long pathways for electrons at the electrode. Both SFCN and Pr_0.8_Sr_1.2_(Co,Fe)_0.8_Nb_0.2_O_4+δ_/CoFe alloy have exsolved metal under reduction indicating that designing a material with exsolved metal under reduction will help to achieve a high conductivity in a reducing atmosphere. The exsolved metal may also improve the catalytic activity under the fuel cell operating conditions^34^,^35^.

## Fuel Cell Performance when SrFe_0.8_Cu_0.1_Nb_0.1_O_3−δ_ Was Used as the Anode

**Figure 4:**
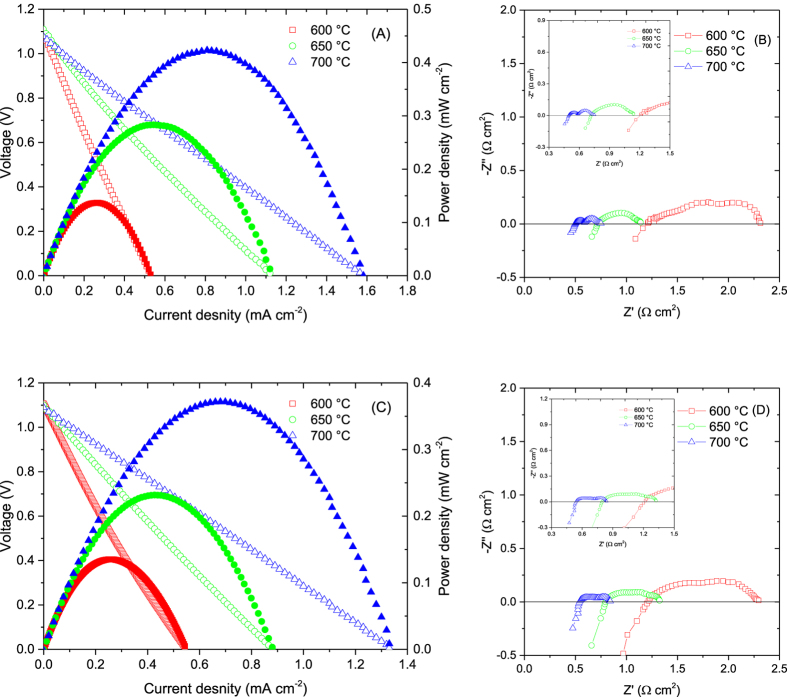
Fuel cell performance. Current-voltage curves (**A,C**) and impedance spectra (**B,D**) of solid oxide fuel cells with SrFe_0.8_Cu_0.1_Nb_0.1_O_3−δ_ (**A,B**) and SrFe_0.9_Nb_0.1_O_3−δ_ (**C,D**) anode.

Solid oxide fuel cells with a SrFe_0.8_Cu_0.1_Nb_0.1_O_3−δ_ anode, YSZ electrolyte and La_0.8_Sr_0.2_FeO_3−δ_ cathode were fabricated. The performance and the corresponding A.C. impedance spectra of hydrogen/air fuel cell at different temperatures are shown in Fig. 4A,B. Good fuel cell performance with a power density of 423 mW/cm^2^ was observed at 700 °C. The total polarisation resistance of the electrode at 700 °C was only 0.25 Ω cm^2^ indicating SrFe_0.8_Cu_0.1_Nb_0.1_O_3−δ_ performs well as an anode for SOFCs. However, a small amount of Ru/CeO_2_ was introduced to improve the catalytic activity. For comparison, a SOFC with the same electrolyte and cathode was assembled with the Cu-free anode SrFe_0.9_Nb_0.1_O_3−δ_ used instead of the Cu doped anode. As shown in Fig. 4C,D, the highest power density at 700 °C was 372 mW/cm^2^ which was slightly lower than that for a SOFC with SrFe_0.8_Cu_0.1_Nb_0.1_O_3−δ_ anode. The total electrode polarisation was 0.30 Ω cm^2^ at 700 °C. The series resistance was 0.54 Ω cm^2^ which is also slightly higher than that of the cell where the SrFe_0.8_Cu_0.1_Nb_0.1_O_3−δ_ anode was used (0.50 Ω cm^2^) (Fig. 4B,D). This indicates that the high conductivity of SrFe_0.8_Cu_0.1_Nb_0.1_O_3−δ_ reduces both series and electrode polarisation resistances leading to high fuel cell performance. It should be noted that the exsolved Fe (or Fe rich FeCu alloy) at the SrFe_0.8_Cu_0.1_Nb_0.1_O_3−δ_ anode may also improve the anode catalytic activity resulting in lower electrode polarisation resistance. The Arrhenius plot area-specific resistances (ASRs) for non-ohmic resistance of SrFe_0.9_Nb_0.1_O_3−δ_ and SrFe_0.8_Cu_0.1_Nb_0.1_O_3−δ_ anodes are shown in Figure S6. At 600 °C, the ASR for the two oxide anodes are similar but the ASR for SrFe_0.8_Cu_0.1_Nb_0.1_O_3−δ_ is much lower at 650 and 700 °C indicating better catalytic activity. Copper, copper alloy and iron alloy has been widely studied as excellent anode catalysts in SOFCs^6^,^36^,^37^,^38^,^39^. In an early reported, it was found that FeCo alloy was exsolved from Pr_0.4_Sr_0.6_Co_0.2_Fe_0.7_Nb_0.1_O_3−δ_ anode while excellent fuel cell performance has been achieved although the conductivity of this material was not presented^40^.

## Conclusions

In this work, a conductive perovskite oxide SrFe_0.8_Cu_0.1_Nb_0.1_O_3−δ_ has been identified which exhibits the highest conductivity in a reducing atmosphere for oxides which also exhibit a high conductivity in air. This high conductivity is probably related to the exsolution of Fe (or Fe-rich FeCu alloy) during the high temperature reducing process. After the reduction, besides the exsolved metal, the major phase is that of the perovskite. Good performance at intermediate temperatures in solid oxide fuel cells using the SrFe_0.8_Cu_0.1_Nb_0.1_O_3−δ_ based anode indicates that it is a promising anode for SOFCs. This study indicates that the conductivity of the oxides with exsolved metal is significantly high. This provides a strategy to identify a good material with high conductivity in both air and reducing atmospheres, i.e., starting from an oxide material with high conductivity in air, followed by introducing transition elements such as Fe, Co, Ni, Cu in the lattice which may be exsolved from the lattice on reduction therefore resulting in high conductivity in a reducing atmosphere.

## Methods

### Synthesis of oxides

SrFe_0.9−x_Cu_x_Nb_0.1_O_3−δ_ (x = 0–0.4) were synthesised by a sol-gel process, similar to previously reported processes^20^. A stoichiometric amount of C_4_H_4_NNbO_9_·xH_2_O (99.9%, Sigma Aldrich) was dissolved in distilled water. H_2_O_2_ was added to the niobium solution until a colour change was elicited. Citric acid (99^+^%, Alfa Aesar), in a 2:1 molar ratio to the metal ions in the final product, was added and heated till a solution was formed. Stoichiometric amounts of Sr(NO_3_)_2_ (98%, Alfa Aesar), Fe(NO_3_)_3_·9H_2_O (98%, Alfa Aesar) and Cu(NO_3_)_2_·2.5 H_2_O (ACS grade, Alfa Aesar) were dissolved in distilled water. The solutions were mixed first then heated until gelation. The resultant gel formed was fired at 600 °C for 2 hours and further fired at 1200–1300 °C for 4–24 hours. The as-prepared powders were uniaxially pressed at 221 MPa in to pellets (ø ≈ 13 mm × 2 mm) and subsequently sintered in air at 1200 °C–1450 °C for 4–10 hours. The details are listed in Table S1.

### Analytical Procedures

X-ray data was collected on a PANanalyticalX’Pert Pro in the Bragg-Brentano reflection geometry with a Ni-filtered Cu Kα source (1.5405 Å), fitted with a X’Celerator detector and an Empyrean CuLFF xrd tube. Absolute scans in the 2*θ* range of 5–100° with step sizes of 0.0167° were used during data collection. GSAS^22^ software was used to perform a least squares refinement of the lattice parameters of all the samples.

Thermal analysis was conducted using a Stanton Redcroft STA 1500 Thermal Analyser on heating from room temperature to 800 °C and on cooling from 800 °C to room temperature in air, with a heating/cooling rate of 10 °C/min, and in 5% H_2_/Ar, again with a heating/cooling rate of 10 °C min^−1^, and with a flow rate of 5% H_2_/Ar of 50 mLmin^−1^.

Scanning electron mircroscopy (SEM) measurements were carried out on a ZEISS SUPRA 55-VP Field Emission Scanning Electron Microscope. The densities of the pellets were determined from the measured mass and volume. Theoretical densities were calculated using experimental lattice parameters and the chemical formula SrFe_0.9−x_Cu_x_Nb_0.1_O_3−δ_ (x = 0–0.4). The relative density of the pellets was 80–90% for all compounds.

### Conductivity Testing

The pellets (ø ≈ 13 mm × 2 mm) were coated on opposing sides using silver paste for all samples. The conductivity of the samples was measured primarily in air between 300 °C to 700 °C. Secondary measurements over the same temperature range were conducted in 5% H_2_/Ar following an equilibration step of 10 hours at 700 °C in 5% H_2_/Ar. Measurements were conducted using a pseudo four-terminal DC method using a SolartronCell 1470E electrochemical interface controlled by CellTest software with an applied current of 1 - 0.1 A.

### Fuel Cell Fabrication and Testing

The electrolyte support cell used in this study was prepared through a tape casting process, with the outer two layers having pore formers. A dense YSZ slurry was prepared by mixing YSZ powder with methyl ethyl ketone and ethanol, and along with binders (polyvinyl butyral and polyethylene Glycol). Porous YSZ was prepared by adding YSZ powder with methyl ethyl ketone and ethanol, binders (polyvinyl butyral and polyethylene Glycol) and graphite (UCP-2 grade, Alfa Aesar) sequentially. The resultant two slurries were tape-casted separately. The porous–dense–porous YSZ structure was prepared by laminating three green tapes, followed by sintering at 1500 °C for 4 h, after which the porosity was approximately 65%. The final thicknesses of the dense electrolyte and porous electrode were ~100 μm and 45 μm, respectively. The diameter of the porous YSZ region was 0.67 cm. To prepare composites of SFN-YSZ and SFCuN-YSZ electrolyte supported cell, the precursor solutions were firstly prepared by dissolving nitrate salts of Sr, Fe, Cu, and Nb in distilled water with the addition of quantitative amounts of citric acid. The SFCuN -YSZ anode was prepared by infiltrating the precursor aqueous solution into the anode side of the three-layered YSZ backbone. SFCuN was infiltrated into then porous YSZ backbone by a multi-step process followed by heating at 450 °C to decompose nitrates and citric acid. The infiltration process was repeated until 40 wt% loading of the oxide was achieved. Finally, SFCuN-YSZ anode wafers were calcined in air at 1000 °C. The LSF (La_0.8_Sr_0.2_FeO_3−δ_)-YSZ cathode was fabricated by infiltration using an aqueous solution of nitrate salts of La, Sr and Fe on a porous YSZ backbone opposite the anode layer. This was then calcined in air at 850 °C. 2 wt% Ru and 10 wt% ceria were also infiltrated into the anode and heated in air at 450 °C. For fuel cell performance tests, the cells were mounted on alumina tubes with ceramic adhesives (Ceramabond 552, Aremco). Ag paste and Ag wire were used for the electrical connections to both the anode and the cathode. The entire cell was placed inside a furnace and heated to the desired temperature. V–i polarization curves were measured using a Potentiostat in the temperature range of 700–600 °C. The fuel cell performance was measured by a Solartron 1470e Electrochemical Interface coupled with a Solartron 1455 controlled by electrochemical software Solartron CellTest. The a.c. impedance was measured in the frequency range between 1 MHz and 0.01 Hz at the amplitude of the a.c. signal 20 mV.

## Additional Information

**How to cite this article**: Lan, R. *et al*. A perovskite oxide with high conductivities in both air and reducing atmosphere for use as electrode for solid oxide fuel cells. *Sci. Rep.* **6**, 31839; doi: 10.1038/srep31839 (2016).

## Supplementary Material

Supplementary Information
