# Supplementary material for: A perovskite oxide with high conductivities in both air and reducing atmosphere for use as electrode for solid oxide fuel cells
Source: Sci Rep. 2016 Aug 22;6:31839. doi: 10.1038/srep31839 (PMC4992832; doi:10.1038/srep31839)
Supplement: Supplementary Information [file srep31839-s1.pdf]

Supplementary Information for

A perovskite oxide with high conductivities in both air and reducing atmosphere for use as electrode for solid oxide fuel cells

Rong Lan [1], Peter I. Cowin [3], Sivaprakash Sengodan [1] and Shanwen Tao [1,2]*

[1] School of Engineering, University of Warwick, Coventry CV4 7AL, UK
[2] Department of Chemical Engineering, Monash University, Clayton, Victoria 3800, Australia
[3] Department of Chemical and Process Engineering, University of Strathclyde, Glasgow G1 1XJ, UK

*Corresponding author. S.Tao.1@warwick.ac.uk

Supplementary Table S1: Firing and sintering conditions for $SrFe_{0.9-x}Cu_xNb_{0.1}O_{3-\delta}$ (x = 0 – 0.4)

Compound	Firing conditions	Sintering conditions
$SrFe_{0.9}Nb_{0.1}O_{3-\delta}$	1300 °C, 5 hours	1450 °C, 5 hours
$SrFe_{0.8}Cu_{0.1}Nb_{0.1}O_{3-\delta}$	1300 °C, 15 hours	1350 °C, 5 hours
$SrFe_{0.7}Cu_{0.2}Nb_{0.1}O_{3-\delta}$	1300 °C, 10 hours	1300 °C, 5 hours
$SrFe_{0.6}Cu_{0.3}Nb_{0.1}O_{3-\delta}$	1300 °C, 5 hours	1300 °C, 5 hours
$SrFe_{0.5}Cu_{0.4}Nb_{0.1}O_{3-\delta}$	1200 °C, 24 hours	1200 C, 10 hours

Supplementary Table 2: 'Goodness of fit' parameters, lattice parameters and atomic parameters from GSAS refinement of $SrFe_{0.9-x}Cu_xNb_{0.1}O_{3-\delta}$ (x = 0 - 0.5) after synthesis in air.

		$SrFe_{0.9}Nb_{0.1}O_{3-\delta}$	$SrFe_{0.8}Cu_{0.1}Nb_{0.1}O$	$SrFe_{0.7}Cu_{0.2}Nb_{0.1}O_3$	$SrFe_{0.6}Cu_{0.3}Nb_{0.1}O_3$	$SrFe_{0.5}Cu_{0.4}Nb_{0.1}O_3$
χ^2		2.775	2.607	5.116	2.541	1.922
Rp (%)		5.21	5.553	7.09	5.44	4.92
wRp (%)		3.76	4.00	4.82	4.05	3.87
Space		*Pm-3m*	*Pm-3m*	*Pm-3m*	*Pm-3m*	*Pm-3m*
a (Å)		3.8789(3)	3.8817(1)	3.8889(9)	3.8939(1)	3.8933(7)
V (Å^3)		58.36(1)	58.49(1)	58.81(4)	59.04(1)	59.01(3)
Sr	x	0	0	0	0	0
	y	0	0	0	0	0
	z	0	0	0	0	0
	U_{is}	0.006(1)	0.001(1)	0.001(1)	0.005(1)	0.001(1)
Fe/Cu/N	x	0.5	0.5	0.5	0.5	0.5
	y	0.5	0.5	0.5	0.5	0.5
	z	0.5	0.5	0.5	0.5	0.5
	U_{is}	0.008(1)	0.015(1)	0.017(1)	0.019(1)	0.008(1)
O	x	0	0	0	0	0
	y	0.5	0.5	0.5	0.5	0.5
	z	0.5	0.5	0.5	0.5	0.5
	U_{is}	0.011(1)	0.022(1)	0.055(2)	0.020(1)	0.032(1)

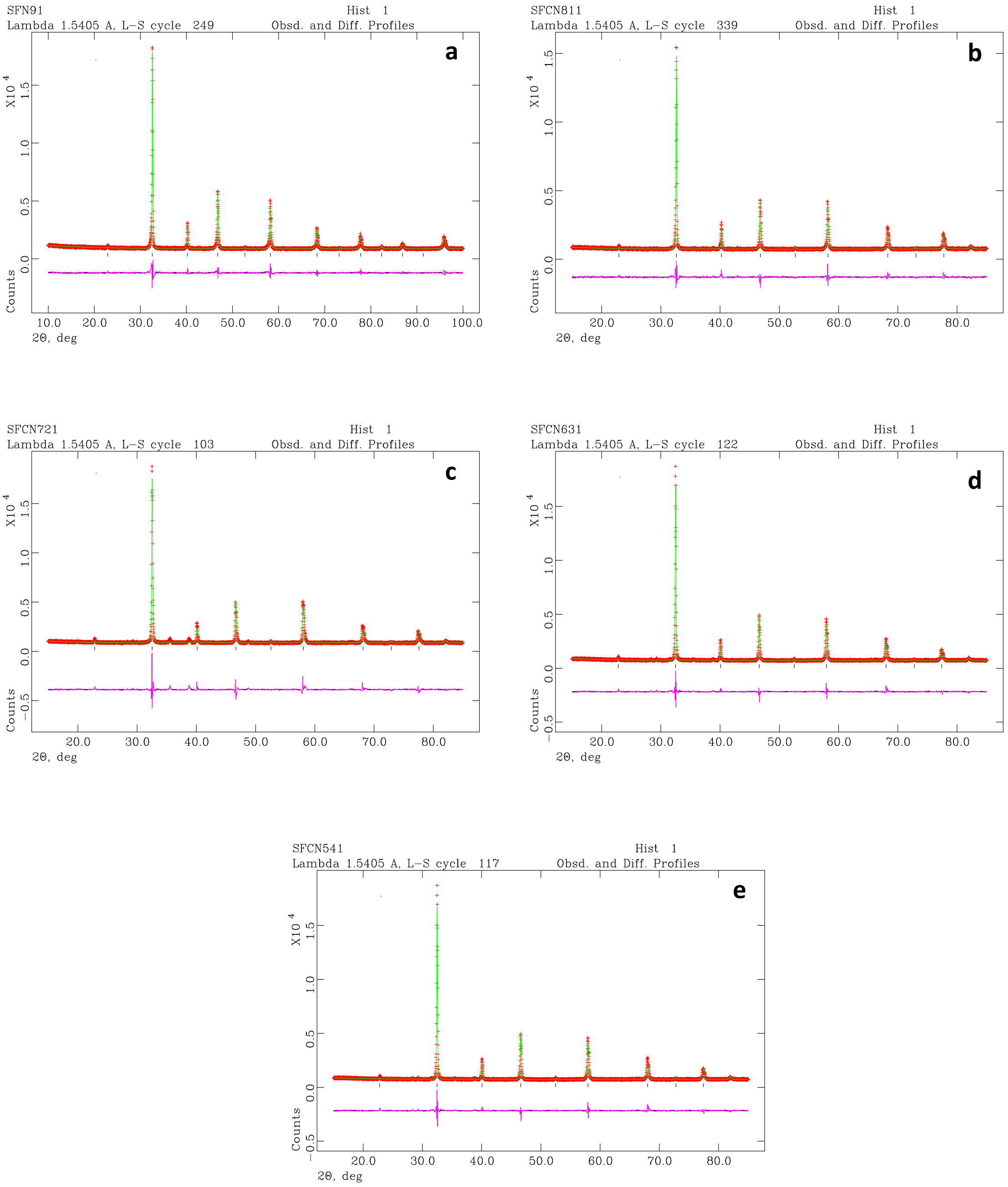

Supplementary Figure 1: GSAS plots for $SrFe_{0.9-x}Cu_xNb_{0.1}O_{3-\delta}$, x = 0 (a), x = 0.1 (b), x = 0.2 (c), x = 0.3 (d), x = 0.4 (e) obtained in air.

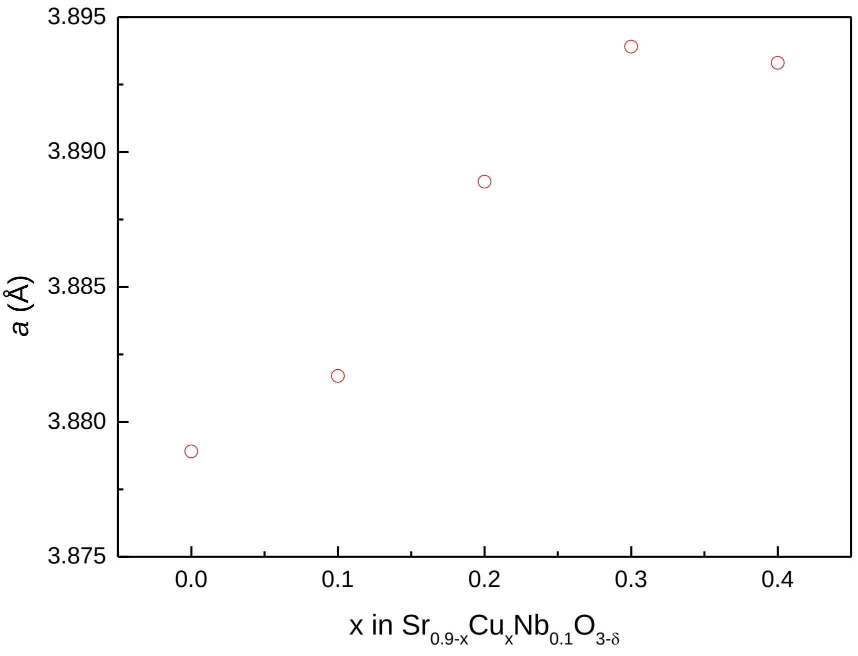

Supplementary Figure 2: Variation of lattice parameters for $SrFe_{0.8}Cu_{0.1}Nb_{0.1}O_{3-\delta}$ (x = 0-0.4)

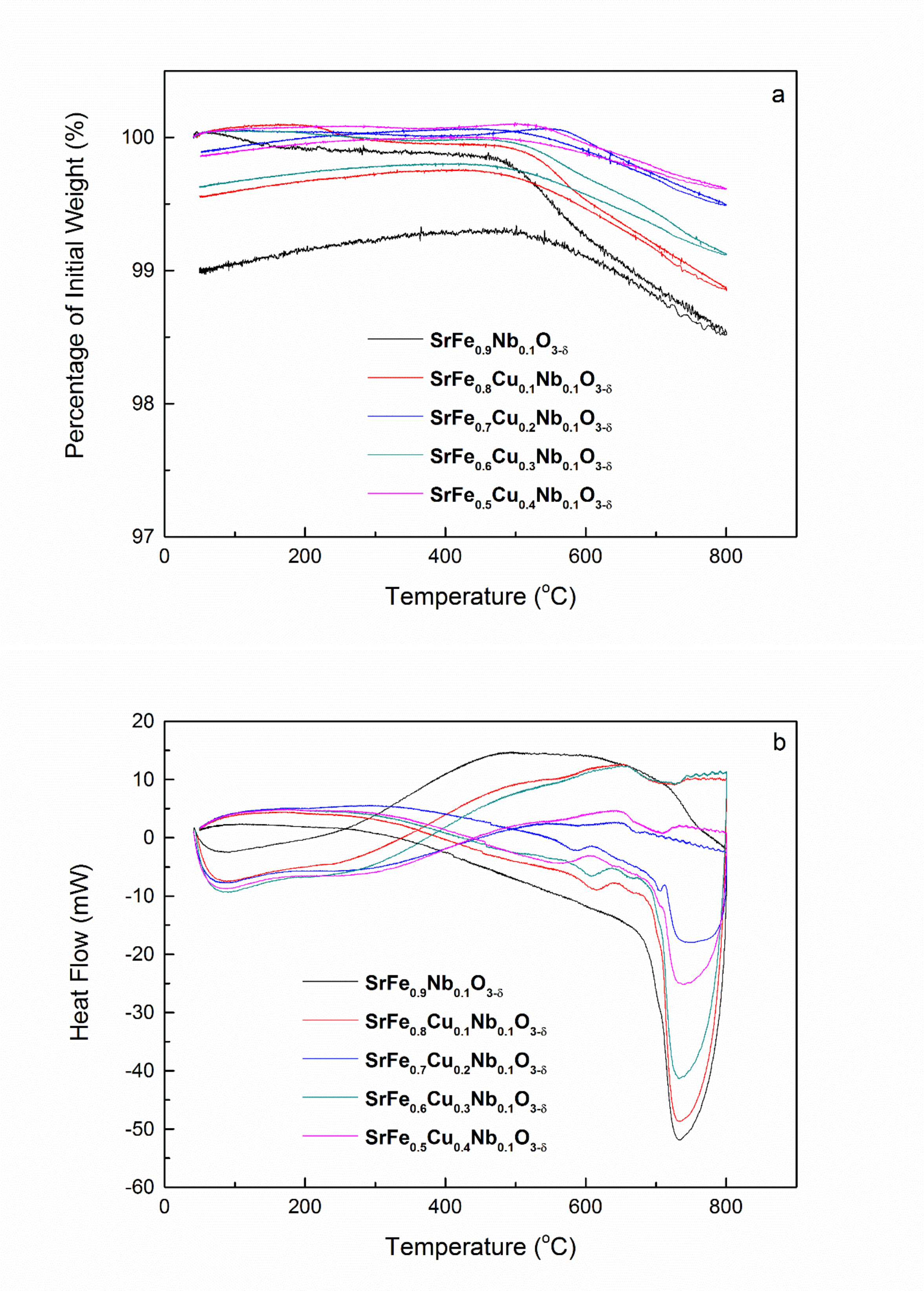

Supplementary Figure 3: Thermogravimetric analysis (a) and differential scanning calorimetry (b) of $SrFe_{0.8}Cu_{0.1}Nb_{0.1}O_{3-\delta}$ (x = 0-0.4) in air

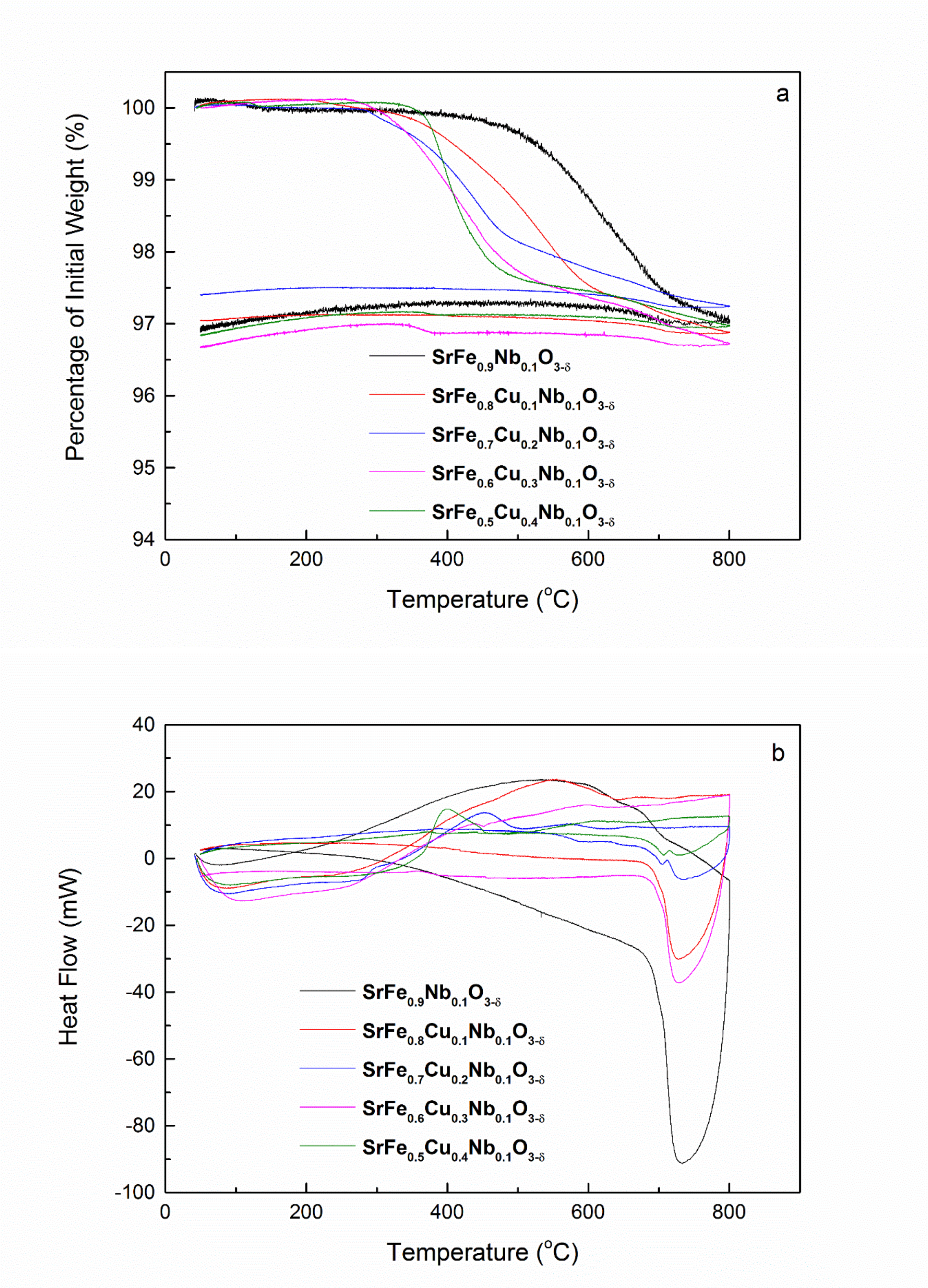

Supplementary Figure 4: Thermogravimetric analysis (a) and differential scanning calorimetry (b) of $SrFe_{0.8}Cu_{0.1}Nb_{0.1}O_{3-\delta}$ (x = 0-0.4) in 5% H_2/Ar

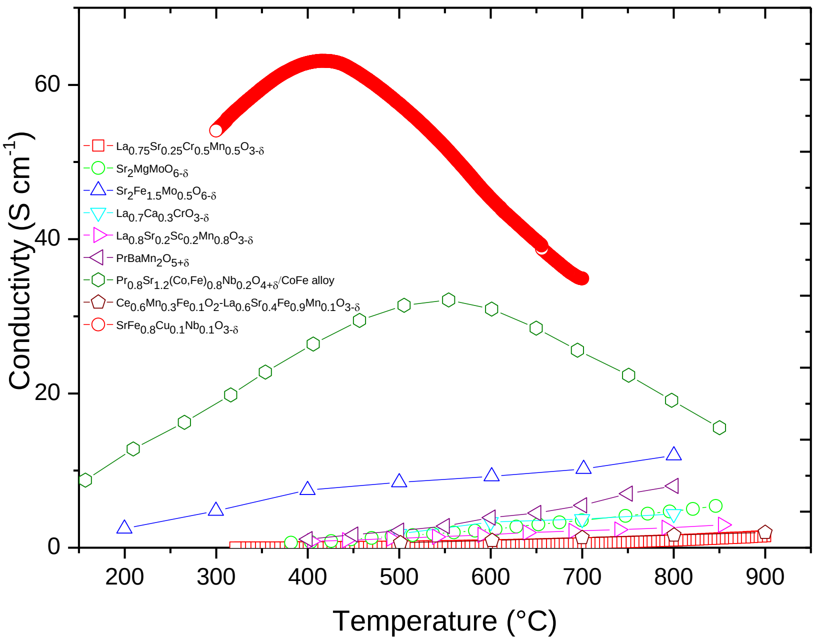

Supplementary Figure 5: Comparison of conductivity of reported anode materials for symmetrical solid oxide fuel cells at different temeprature. * $SrFe_{0.8}Cu_{0.1}Nb_{0.1}O_{3-\delta}$, this study.

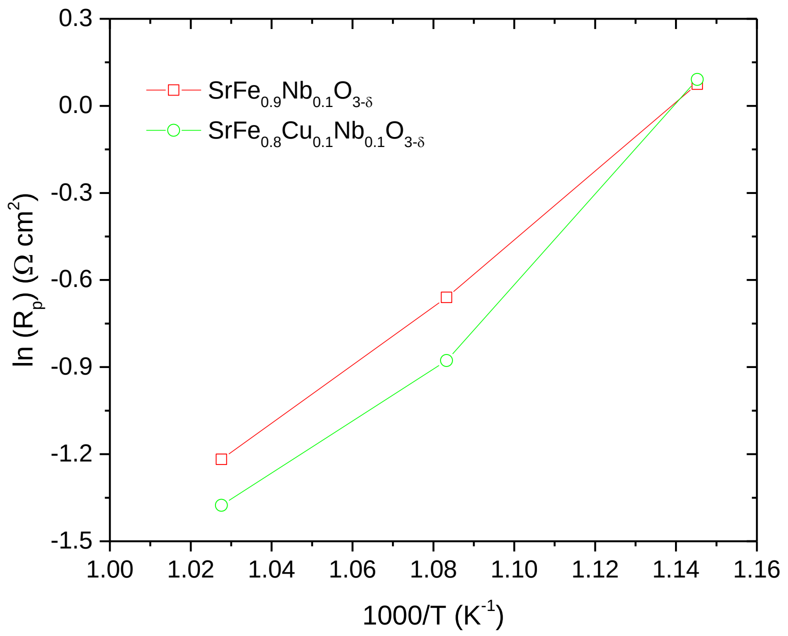

Supplementary Figure 6: Arrhenius plot for non-ohmic resistance of $SrFe_{0.9}Nb_{0.1}O_{3-\delta}$ and $SrFe_{0.8}Cu_{0.1}Nb_{0.1}O_{3-\delta}$ anode.
